# Integrin Crosstalk Contributes to the Complexity of Signalling and Unpredictable Cancer Cell Fates

**DOI:** 10.3390/cancers12071910

**Published:** 2020-07-15

**Authors:** Ivana Samaržija, Ana Dekanić, Jonathan D. Humphries, Mladen Paradžik, Nikolina Stojanović, Martin J. Humphries, Andreja Ambriović-Ristov

**Affiliations:** 1 Laboratory for Cell Biology and Signalling, Division of Molecular Biology, Ruđer Bošković Institute, 10000 Zagreb, Croatia; Ivana.Samarzija@irb.hr (I.S.); Mladen.Paradzik@irb.hr (M.P.); Nikolina.Stojanovic@irb.hr (N.S.); 2 Laboratory for Protein Dynamics, Division of Molecular Medicine, Ruđer Bošković Institute, 10000 Zagreb, Croatia; Ana.Dekanic@irb.hr; 3 Wellcome Centre for Cell-Matrix Research, Faculty of Biology, Medicine & Health, University of Manchester, Manchester M13 9PT, UK; J.D.Humphries@manchester.ac.uk (J.D.H.); Martin.Humphries@manchester.ac.uk (M.J.H.)

**Keywords:** integrin, integrin adhesion complexes, integrin crosstalk, integrin switching, cancer

## Abstract

Integrins are heterodimeric cell surface receptors composed of α and β subunits that control adhesion, proliferation and gene expression. The integrin heterodimer binding to ligand reorganises the cytoskeletal networks and triggers multiple signalling pathways that can cause changes in cell cycle, proliferation, differentiation, survival and motility. In addition, integrins have been identified as targets for many different diseases, including cancer. Integrin crosstalk is a mechanism by which a change in the expression of a certain integrin subunit or the activation of an integrin heterodimer may interfere with the expression and/or activation of other integrin subunit(s) in the very same cell. Here, we review the evidence for integrin crosstalk in a range of cellular systems, with a particular emphasis on cancer. We describe the molecular mechanisms of integrin crosstalk, the effects of cell fate determination, and the contribution of crosstalk to therapeutic outcomes. Our intention is to raise awareness of integrin crosstalk events such that the contribution of the phenomenon can be taken into account when researching the biological or pathophysiological roles of integrins.

## 1. Introduction

**Figure 1:**
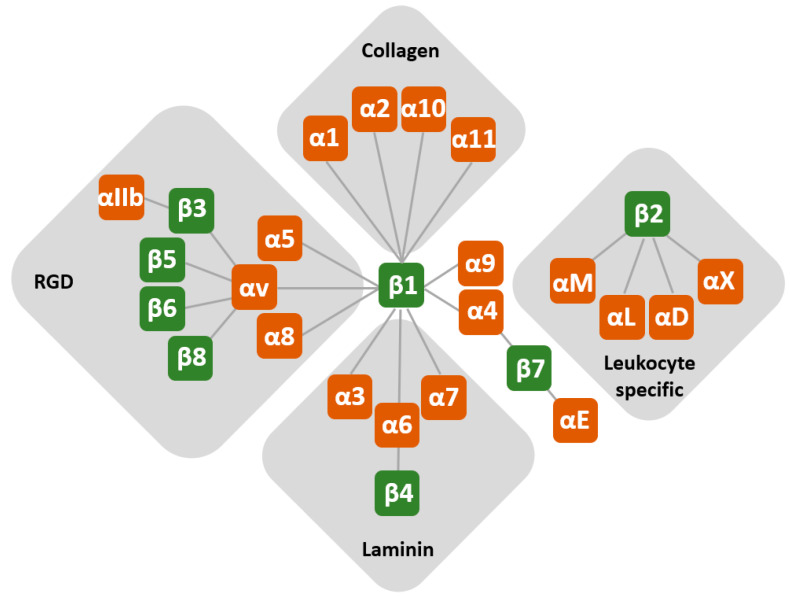
Pairing of integrin subunits. Integrins are transmembrane proteins that mediate cell adhesion to extracellular matrix proteins or cell surface counter-receptors. They are expressed on the cell surface as heterodimers consisting of α and β subunits. To date, 18 different α and 8 β subunits have been identified, which give rise to 24 heterodimers. Different integrins bind different ECM proteins and/or cell surface molecules, which have a specific spatial and temporal distribution pattern in a given tissue. Integrins are separated into subsets of closely related subunits.

Integrins are a large family of ubiquitously expressed transmembrane glycoprotein receptors that function as major sensors of the extracellular environment and regulate many aspects of cell behaviour. The majority of integrins are involved in cell–extracellular-matrix (ECM) interactions, while some of them participate in cell–cell interactions. Upon integrin ligand binding, signals are initiated that reorganise the cytoskeletal networks (actin, microtubules and intermediate filaments) and regulate survival, proliferation, migration and differentiation [1,2,3,4,5,6]. Structurally, integrins are heterodimers and, in humans, 18 different α and eight β subunits have been identified, which give rise to 24 heterodimers (Figure 1). Different integrins bind different ECM proteins and/or cell surface molecules that have a specific spatial and temporal distribution pattern in a given tissue. Every cell type in the body possesses its own specific integrin profile, which is perturbed in different pathophysiological conditions, especially in cancer [3,7,8,9,10]. Upon integrin binding and clustering, proteins are recruited to their cytoplasmic tails to form multimolecular integrin adhesion complexes (IACs), the composition of which has been termed the adhesome. Excellent reviews have been published describing integrin structure and function, as well as IAC composition [3,4,5,11,12,13,14,15,16,17,18,19,20,21]. These reviews also highlight the versatility of integrin family and its contribution to different aspects of cell behaviour. Consequently, the precise and dynamic regulation of the expression of a single integrin type has the potential to directly affect cell signalling and fate.

Functional and morphological analyses have defined several major forms of IACs, including nascent adhesions (NAs), focal adhesions (FAs), fibrillar adhesions (FBs) [16,22], hemidesmosomes (HDs) [21] and recently discovered reticular adhesions (RAs) [13]. NAs are small, transient structures, turning over in order of minutes, sampling the local ECM before disassembling or moving on to form more stable, mature FA structures which are strongly associated with actin filaments [12,23]. FBs are long, stable structures that run parallel to bundles of fibronectin *in vivo* and are highly enriched in tensin and α5β1 integrin [24,25]. HDs are multiprotein complexes that enable the stable adhesion of basal epithelial cells’ internal keratin intermediate filament network to the underlying basement membrane and have a different molecular composition from FAs and FBs [21]. Finally, RAs are new class of IACs that normally lack association with the cytoskeleton, are rich in components of the clathrin-mediated endocytosis machinery and are also termed clathrin-coated plaques, flat clathrin lattices, or clathrin sheets [13,26].

Schwartz and Ginsberg [27] defined the term ”crosstalk” between integrins or between integrins and growth factor receptors as ”unwanted signals in a communication channel caused by the transfer of energy from another circuit” that may lead to unpredictable and potentially deleterious biological responses. The same term integrin crosstalk was also used by Gonzales and colleagues [28] for a mechanism in which one integrin regulates the activation state of a different integrin in the same cell. They also utilised the term ”transdominant inhibition”, which has been used by other researchers [29,30]. Here, we review the evidence for the modulation of activation/expression of one integrin affecting the activation/expression of another integrin and we use the umbrella term of integrin crosstalk. Since blocking antibodies and inhibitors, as well as the manipulation of integrin subunit expression, either by overexpression, knockdown or knockout, is widely used in research and could potentially be translated *in vivo* and into the clinic, our aim is to review integrin crosstalk events that may lead to unpredictable biological responses. We collected integrin crosstalk data from many systems, and we believe this overview provides a useful source of information for researchers that are interested in integrin activation/expression in their experimental settings. It should be noted that most of the data collected describe integrin crosstalk in cancer as this is the focus of our own research. We also emphasize the potential clinical implications of this phenomenon.

## 2. Regulation of Integrin Expression

The individual integrin α or β subunits are not expressed on the cell surface. Only those assembled as heterodimers, the process which occurs in the endoplasmic reticulum (ER), are displayed on the cell surface and are able to bind their ligands and trigger signalling [31]. Their transport from ER to the plasma membrane is allowed only if they attain their native structure [32,33]. Ca^2+^ has a crucial role in integrin folding, assembly and trafficking maintaining the receptors in an inactive form until they reach the cell surface [34]. When expressed on the cell surface, integrin heterodimers can exist in three different states (which equate to conformational classes): (i) an inactive form with low affinity for ligand; (ii) a primed form with high affinity for ligand or (iii) a fully activated ligand-bound form [35,36]. Integrin activation is bidirectional. In “inside-out“ signalling, integrins are activated by conformational changes due to the binding of talin and kindlin to the cytoplasmic tail of integrins [37,38]. Integrins that bind with high affinity ligands trigger ”outside-in” signals [31,39]. The conformational changes that accompany inside-out and outside-in signalling are very similar, emphasising the role of integrins in relaying mechanochemical information between the cytoplasm and plasma membrane.

Because of their essential role in the cell, the expression of integrin heterodimers on the cell surface is precisely and dynamically regulated on several levels by a multitude of mechanisms including: (i) regulation of integrin protein levels by transcriptional or post-transcriptional mechanisms; (ii) alteration of integrin protein primary sequence by alternative splicing of mRNA; (iii) mobilization to the cell surface of pre-existing intracellular stores of integrins; and (iv) modulation of integrin internalisation and recycling. Initially, it was shown that ECM controls the expression of integrin subunits and that this regulation is exerted at both transcriptional and post-transcriptional levels [40]. Subsequently, many signalling pathways and signalling molecules, such as growth factors, cytokines, hormones and pharmacological agents, as well as microRNAs, have been shown to regulate integrin expression in a myriad of cell types, both on mRNA and protein level [41,42,43,44,45]. Alternative splicing has been found for mRNAs of several integrins during development and tumorigenesis and was shown to be tissue-specific [41]. Although the expression of particular integrin subunits is regulated by different mechanisms, it has been shown via knockin, knockdown and knockout experiments that the repertoire of integrin heterodimers on the cell surface depends on the availability of both α and β integrin subunits in the intracellular reservoirs. However, the heterodimeric nature of integrins, the fact that many heterodimers share the same α or β subunits and the limited data on the regulation of expression of αβ integrin heterodimers sharing the same α or β subunits makes the conclusion about the mechanisms of the pairing hierarchy difficult. For example, it has been shown in WM-266-4 melanoma cells that the number of αvβ3 and αvβ5 heterodimers on the cell surface depends on the level of expression of β3 and β5 subunits, respectively, but the expression of the αv gene dictates the number of αvβ1 heterodimers [46]. Similarly, in human lung fibroblasts WI-38 β1 subunit is made in excess over α subunits, and assembly of β1 subunits with rate-limiting α subunits is required for expression of αβ1 on the cell surface [47]. Finally, integrin expression can be regulated by modulation of integrin internalisation and recycling, which has been shown to contribute to the dynamic remodelling of adhesion [48].

## 3. The Integrin Repertoire Is Changed or ”Switched” in Cancer Cells—an Indication of Integrin Crosstalk Events

Given the essential role of integrins in several key cell behaviours and processes, like migration, adhesion, proliferation, survival, differentation, positioning, metabolism, matrix assembly, gene expression and receptor tyrosine kinase signalling, it is no surprise that integrins represent important players during cancer initiation, progression and metastasis [2,5,49,50,51,52,53,54]. Several other important cancer traits, like stemness and drug resistance, as well as the control of tumor microenviroment, have also been shown to be regulated by integrins [2,5,7,17,50,55,56]. 

Oncogenic transformation, directly or indirectly, changes the repertoire of integrins on the surface of a cancer cell. Virtually, the integrin repertoire is ”switched” to support cancer initiation and progression [17,57]. In order to avoid confusion, here we use the term ”integrin switching” as a term for integrin repertoire change on the cell surface, which may or may not be due to ”integrin crosstalk”. 

**Figure 2:**
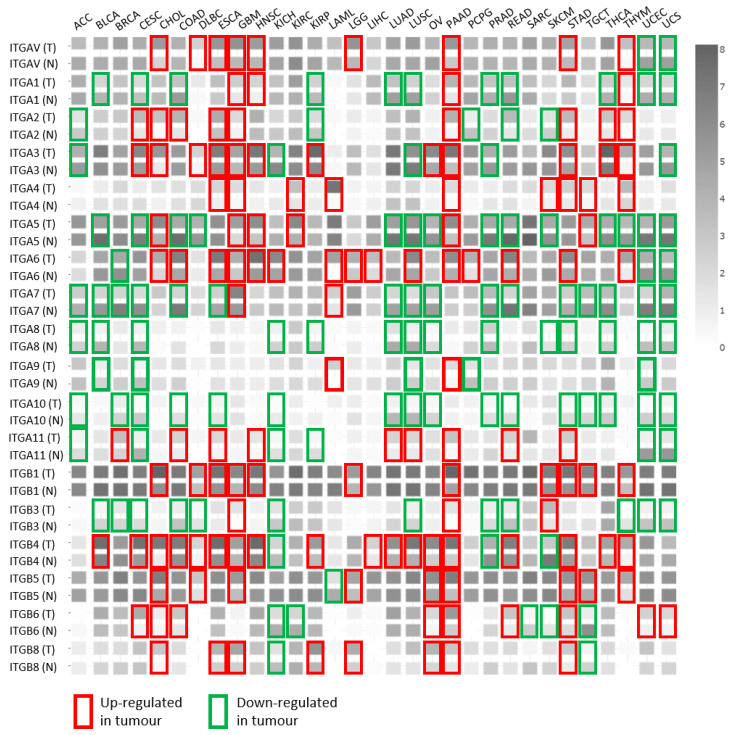
Integrin expression in different tumor (T) and corresponding normal (N) tissues. Statistically significant changes are marked with a rectangle (log2 fold change > 1, *p*-value < 0.01). Figure is made using GEPIA software (http://gepia.cancer-pku.cn/) [58]. Abbreviations and number (in parenthesis) of tumor and normal samples analysed (combined TCGA and GTEx), respectively. Tumors are listed in an alphabetical order: ACC, adrenocortical carcinoma (77, 128); BLCA, bladder urothelial carcinoma (404, 28); BRCA, breast invasive carcinoma (1085, 291); CESC, cervical squamous cell carcinoma and endocervical adenocarcinoma (306, 13); CHOL, cholangiocarcinoma (36, 9); COAD, colon adenocarcinoma (275, 349); DLBC, diffuse large B-cell lymphoma (47, 337); ESCA, esophageal carcinoma (182, 286); GBM, glioblastoma multiforme (163, 207); HNSC, head and neck squamous cell carcinoma (519, 44); KICH, kidney chromophobe (66, 53); KIRC, kidney renal clear cell carcinoma (523, 100); KIRP, kidney renal papillary cell carcinoma (286, 60); LAML, acute myeloid leukemia (173, 70); LGG, lower grade glioma (518, 207); LIHC, liver hepatocellular carcinoma (369, 160); LUAD, lung adenocarcinoma (483, 347); LUSC, lung squamous cell carcinoma (486, 338); OV, ovarian serous cystadenocarcinoma (426, 88); PAAD, pancreatic adenocarcinoma (179, 171); PCPG, pheochromocytoma and paraganglioma (182, 3); PRAD, prostate adenocarcinoma (492, 152); READ, rectum adenocarcinoma (92, 318); SARC, sarcoma (262, 2); SKCM, skin cutaneous melanoma (461, 558); STAD, stomach adenocarcinoma (408, 211); TGCT, testicular germ cell tumors (137, 165); THCA, thyroid carcinoma (512, 337); THYM thymoma (118, 339); UCEC, uterine corpus endometrial carcinoma (174, 91); UCS, uterine carcinosarcoma (57, 78).

As we already emphasized, the transcription of integrin subunits does not determine the repertoire of integrin heterodimers on the cell surface but can serve as an indication of the integrin “switch“. Therefore, we analyzed the expression of integrin subunits across different cancer types and corresponding normal tissues using the TCGA (https://www.cancer.gov/) and GTEx databases (https://www.gtexportal.org/) using GEPIA software [58]. As shown in Figure 2, mRNA expression of integrin subunits is highly perturbed in different cancer types. In glioblastoma and pancreatic adenocarcinoma, the upregulation of almost all integrin subunits is observed. Conversely, in sarcoma, only the downregulation of integrin β6 is found. The shade of grey in Figure 2 indicates the level of expression of a particular integrin subunit, showing that integrin subunits α3, α5, α6, αv, β1, β4 and β5 are the most highly expressed. The combination of these integrin subunits give rise to the integrin heterodimers α3β1, αvβ1, αvβ5, α5β1 and α6β4 shown to be involved in formation of FAs (α3β1, αvβ1, αvβ5), FBs (α5β1) [12,25]. They are also highly altered in 31 cancer tissues analysed here compared to normal ones. Integrins β1 and β5 were found to be exclusively upregulated, in 11 and nine cancers, respectively. Integrin αv was upregulated in nine and downregulated only in two cancer types, while integrin α3 was found to be upregulated in 12 and downegulated in five. The integrin α5 which is the only one forming FBs is found to be more frequently downregulated (in 12 tumours) than upregulated (in six). However, the most pronounced changes were observed for integrin subunit α6 that was upregulated in 18 and downregulated in three, and β4 that was upregulated in 15 and downregulated in three cancer types, respectively. These particular integrin subunits heterodimerise into α6β4 which form HDs [21]. Therefore, the changes observed are expected, since most of the cancer types analysed in Figure 2 are carcinomas developed from epithelial tissues for which the importance of hemidesmosome components in carcinogenesis was recently reviewed [59]. Moreover, recently published data by Wang and colleagues [14] revealed a novel role for HDs as regulators of cellular mechanical forces. They showed the existence of a mechanical coupling between different IACs, specifically HDs and FAs and RAs.

As we emphasized before, integrins must form heterodimers in order to be expressed on the cell surface and, therefore, the expression of integrin transcripts is not a firm indication of the protein expression on the cell surface. In accordance with this, discrepancies between the expression of integrin subunit mRNA and protein in particular cancer tissues have been found. For example, mRNAs of integrin subunits α5, α7, β3, β6 and β8 are underexpressed in prostate cancer tissue in comparison to normal (Figure 2), while the immunohistochemistry data suggest their overexpression [60]. One of the possible explanations for this might be that immunohistochemistry data does not discriminate between integrins expressed on the cell surface as heterodimers from integrin subunits in the intracellular stores. An example of how expression of integrin subunit transcripts altered in tumors does not correlate with their involvement in the biology of particular cancer can be found in melanoma. Namely, the expression of integrin subunits β1, β3 and α4 is increased while expression of α2, α5, α8, β4 and β6 is decreased as compared to normal tissue (Figure 2). However, increased expression of integrins α5β1 and αvβ3 results in a poor melanoma prognosis, increased cell invasion, and metastasis [61,62], while data obtained *in vitro* in different melanoma cell lines showed that integrin αvβ5 is involved in the higly aggressive phenotype of cells expressing neuropilin 1 [63], and is involved in sensitivity to microtubule poison paclitaxel and increased *in vitro* migration and invasion [64,65]. 

## 4. Integrin Crosstalk 

A common response of a cancer cell to anticancer treatment is the activation of diverse compensatory mechanisms (reviewed in [66,67,68]) that are elicited to circumvent the inhibition of a targeted signalling pathway. This kind of plasticity and the ability of the cell to rewire its signalling pathways and internal processess is very often seen in other pathophysiological conditions like nervous system diseases [69] or with host defense pathways [70]. Compensation is based on pathway redundancy, feedback and crosstalk mechanisms [66]. Besides the redundancy in ligand affinity, the integrin family possesses another innate characteristic similar to the cell compensation mechanisms in the way that a change in the expression of integrin subunits or the activation of a certain integrin heterodimer can interfere with the expression or the activation of the other. This phenomenon, that we defined as integrin crosstalk, was described for the first time in 1994 in K562 erythroleukemia cells in which *de novo* expression of αvβ3 led to the inhibition of α5β1 activitation and, consequently, the inhibition of α5β1-mediated phagocytosis [71].

Integrin crosstalk has been mostly observed in experiments *in vitro* in cultured cells of different origin performed in order to show a causal relationship between one integrin heterodimer and either cell adhesion, migration, invasion, phagocytosis, size of particular focal adhesions or transduction efficacy of adenovirus type 5 which uses RGD-binding integrins for internalisation. Interventions in these experiments include integrin subunit overexpression or knockdown, cell exposure to blocking monoclonal antibodies (MoAbs) to prevent integrin signalling or immobilised MoAbs for integrin activation, use of specific integrin inhibitors, seeding cells on specific integrin binding substrates or the introduction of inactive or constitutive active integrin mutants into cells. Table 1 describes integrin crosstalk events collected from the literature. 

A comprehensive analysis of integrin crosstalk in a particular cell model has never been done. Similarly, reviewing the observed events does not indicate the type or frequency of the integrin crosstalk phenomenon. However, by examining Table 1 we can conclude that some integrins are associated in more than one cell type. An apparent inversely proportional relationship in mesenchymal stem cells is found for integrins α1 and α2 [72]. Similarly, *de novo* expression of integrin α3 decreases the activation of αv while α3 blocking antibody activates integrin αvβ3 in several cancer cell types [73]. Integrin crosstalk between integrins α4β1 and αLβ2 has been observed in T cells by several research groups. To be specific, *de novo* expression, the activation or crosslinking of α4β1 leads to activation of αLβ2 and increased migration as a consequence of adhesion of both integrins [74,75,76]. Blocking integrin α4 in oral squamous carcinoma cell line HSC-3 leads to upregulation, while integrin α4 expression leads to the downregulation of both integrins α5 and αv, respectively. The exposure of cells to blocking Abs directed against either integrins α5 or αv, increases α4 and αv or α4 and α5, respectively, indicating that these three integrin α subunits are mutually regulated in a crosstalk fashion [77]. A crosstalk between integrins α5β1 and αvβ3 has been shown in endothelial cells [78,79], glioblastoma U-251MG cells [80], chick chorioallantoic membrane cells (CAMs) [78] and chinese hamster ovary (CHO-B3) cells [81]. In general, integrin α5β1 activation by either binding to a specific substrate or α5β1-specific immobilised MoAb, respectively, increases αvβ3 activation [79,80]. Similarly, the inhibition of α5β1 signalling by an α5β1-blocking MoAb decreases αvβ3 activation [78]. The only exception to the αvβ3 dependency by α5β1 has been shown in CHO cells in which *de novo* expression of α5 decreases αvβ3 activation [81]. Conversely, when integrin αvβ3 was modulated or even depleted in different cell models, the differential effect on α5β1 was observed [71,82,83,84].

The example of integrin crosstalk, which is of outstanding importance to the clinic is the one between different integrin β1 heterodimers and αvβ3. The knockdown of β1 in different human and mouse breast cancer cell lines or kidney cells leads to αvβ3 activation. However, the outcome of these integrin crosstalk events is differential, either increased or decreased metastasis [85,86,87,88,89], indicating that it is very likely that the overall impact on metastasis depends on the strength of the crosstalk between the two integrins. 

Integrin crosstalk is very likely dependent on the repertoire of integrins expressed on the cell and the amount of integrins in intracellular stores. A very simple compensation crosstalk mechanism was observed in melanoma cell line MDA-MB-435S, which preferentially expresses integrins β3 and β5 as αv integrin subunit-binding partners. In this cell line, the knockdown of either subunit β3 or β5 not only downregulates the expression of αvβ3 or αvβ5 but simultaneously upregulates αvβ5 or αvβ3, respectively, maintaining the expression of the similar amount of total integrins αv (both αvβ3 and αvβ5) on the cell surface, representing a certain ”balance” effect. However, a simillar effect was not observed in melanoma RPMI-7951 and breast carcinoma MDA-MB-231 cell lines [64]. Other examples of integrin crosstalk between αv integrins [64,90,91,92] will be discussed in the section dedicated to integrin crosstalk mechanisms.

**Figure 3:**
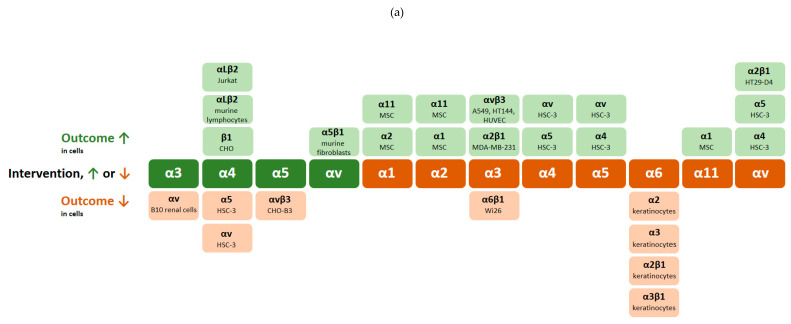

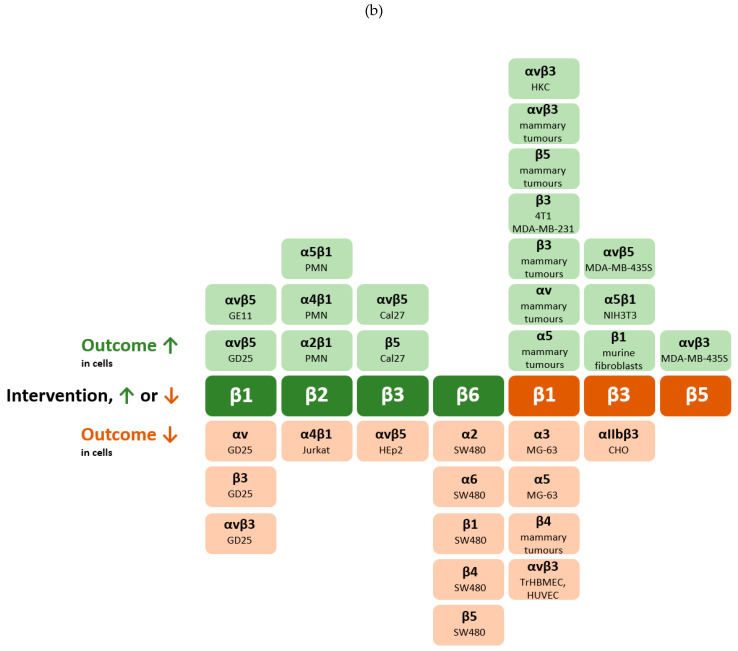

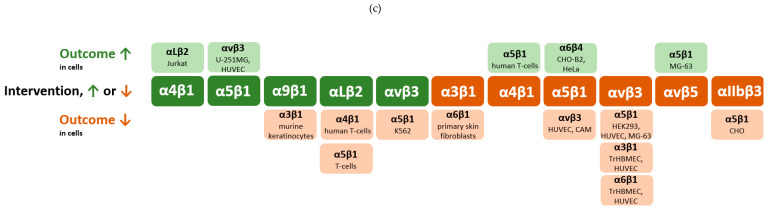
Integrin crosstalk examples documented in experiments that interfere with (**a**) α or (**b**) β integrin subunit or (**c**) integrin heterodimer, listed in Table 1. ↑ denotes upregulation of integrin expression or activation, while ↓ denotes integrin downregulation or inhibition.

Table 1 describes additional integrin crosstalk events observed upon manipulation of integrin subunits or heterodimers in different cell lines. We summarised the integrin crosstalk examples in Figure 3 to show frequency and type of integrin crosstalk derived from the manipulation of either subunits α (Figure 3a), β (Figure 3b) or integrin heterodimers αβ (Figure 3c).

## 5. Molecular Mechanisms of Integrin Crosstalk

**Figure 4:**
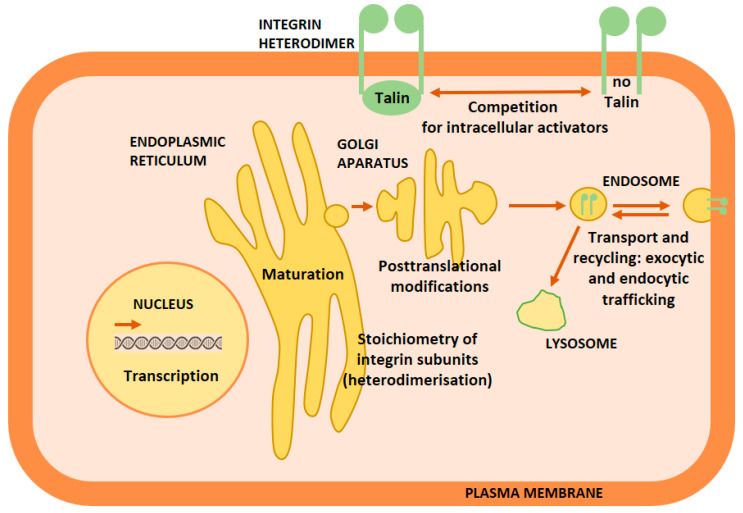
Integrin crosstalk mechanisms. The mechanisms act on several levels: on the level of changes in stoichiometry of integrin subunits, transcription, but also on the level of changes of integrin proteins (maturation, post-translational modifications, transport and recycling) and the activation of an integrin. Integrin crosstalk mechanisms are in lower case.

Mechanisms that trigger integrin crosstalk are diverse and act on several levels of integrin (bio)chemistry (Figure 4). In subsequent sections, we describe the integrin crosstalk mechanisms using examples from the literature. 

### 5.1. Changes in Stoichiometry of Integrin Subunits

As we already emphasized, only integrins assembled as heterodimers are displayed on the cell surface. This process occurs in the ER [31]. Factors affecting the type of heterodimer to be formed are also the propensity of integrin subunits to form certain integrin heterodimers, the relative affinities of integrin subunits for heterodimerization and stoichiometry. Integrin crosstalk is often documented in knockdown and overexpression experiments where the expression of integrin heterodimers is altered by the availibility of integrin subunits. As mentioned in a previous section, in human melanoma cells MDA-MB-435S, knockdown of integrin β3 subunit decreased the amount of integrin αvβ3, but simultaneously upregulated integrin αvβ5, and vice versa. The increased expression of αvβ5 could be a consequence of the liberated amount of integrin αv (upon β3 knockdown) and the excess amount of integrin β5 subunit being able to form αvβ5 and vice versa [64]. These results are in line with results obtained by Koistinen and Heino [46], where they show that the number of αvβ3 and αvβ5 heterodimers on the cell surface depends on the level of the expression of β3 and β5 subunits. We documented a similar mechanism in human laryngeal carcinoma HEp2 cell clones stably transfected with a plasmid coding for integrin subunit β3 that led to *de novo* αvβ3 expression but, in turn, downregulated integrin heterodimer αvβ5. Namely, integrin β3 competed with β5 for the available integrin subunit αv in the cell, which resulted in decreased expression of αvβ5 [90,91]. 

### 5.2. Effects on Transcription

Integrins affect gene expression through numerous mechanisms (crosstalk with catalytically active transmembrane receptors; association with signal transducers; or cytoskeletal-dependent mechanotransduction) and, in some cells, this regulation plays an important role in differentiation, proliferation and other processess in the cell [114]. Among a repertoire of different genes whose expression is affected by integrins are integrins themselves. The effect on transcription was observed in human tongue squamous carcinoma cells Cal27 in which *de novo* expression of integrin subunit β3, that led to *de novo* expression of integrin αvβ3 heterodimer, increased the expression of integrin subunit β5-specific mRNA and integrin heterodimer αvβ5 expression on the cell surface, without transcriptional upregulation of integrin subnit αv [92]. Knockdown of α1, α2 or α11-specific mRNA in human mesenchymal stem cells was shown to upregulate transcription of α2 and α11, α1 and α11 or α1 mRNA, respectively but only α2 or α11 knockdown, preventing interaction with collagen type I, resulted in cell death [72]. Knockdown of α6 mRNA in human keratinocytes decreased transcription of both, α3 and α2 integrin mRNAs [98]. In several papers, using knockdown of integrin β1 in breast carcinoma cells or *in vivo* mouse model of mammary-specific deletion of β1, the increased expression of mRNA specific for β3 was observed [86,87,88,89]. The integrin crosstalk through effects on transcription was also observed in renal cells upon integrin β1 knockdown which increased transcription of β3-specific mRNA [85] and in mouse embryonic fibroblast GD25 cell line in which *de novo* expression of the β1A and β1B decreased the β3 mRNA stability [108].

### 5.3. Effects on Integrin Maturation

The production of functional proteins from translated polypeptides includes enzymatic processing, folding and assembly into oligomeric complexes [115]. The integrin maturation process is shown to be regulated by different molecules like alkaline ceramidase 2 [116], presenilins [117] or low-density lipoprotein receptor-related protein-1 [118]. Integrin crosstalk has been shown at the level of β1 integrin subunit maturation. Koivisto et al. [104] showed that depletion of the pre-β1 integrin subunit pools in ER (during malignant transformation, for example), accelerates the maturation rate of the pre-β1 integrin subunit, which slows down the maturation of the α3 and α5 subunits. Similary, Jaspers and colleagues [96] showed that introducing α4 cDNA and overexpressing this subunit in CHO cells increased the rate of maturation of the β1 precursor and the quantity of β1 integrin on the cell surface.

### 5.4. Changes in Integrin Post-translational Modifications

Post-translational modifications of proteins are diverse and greatly influence protein function and turnover. Recently, it has been shown that N-glycosylation of Asn712 on α5β1 integrin controls the EGFR complex formation with integrin α5β1 or α6β4. The loss of this glycosylation site switched the formation of EGFR-α5β1 complex to EGFR-α6β4, which is known to promote cell growth [103]. Retta and colleaugues [108] observed in mouse embryonic fibroblast GD25 cell line that the *de novo* expression of β1 integrin increased the expression of integrin heterodimer αvβ5 through translational and post-translational effects.

### 5.5. Changes in Transport and Recycling

The endocytic and exocytic trafficking of integrin receptors is an important mechanism regulating their expression on the cell surface and processess they are involved in (reviewed in [48,119]). White et al. [83] showed that recycling of αvβ3 during fibroblast migration antagonizes α5β1 recycling which regulates the balance between persistent and random migration. Interestingly, both active and inactive integrins can be recycled [120].

### 5.6. Competition for Intracellular Activators

Talin is a key regulator of integrin activation [121], and it is not surprising that is an important factor in integrin crosstalk. The example of such a regulation was described in 2004 by Calderwood and colleagues [112] who showed that β3 inhibited the activation of α5β1 through competition with talin, while Gonzalez et al. [105] showed that the β1 integrin subunit negatively modulates αvβ3 integrin–ligand binding via protein kinase A (PKA) and inhibition of protein phosphatase 1 (PP1) activity. The calcium/calmodulin-dependent protein kinase II (CaMKII) is another protein found to be involved in integrin crosstalk, which is activated by integrin α5β1. Ligation of the integrin αvβ3 prevented activation of CaMKII by α5β1, inhibited both phagocytosis and migration mediated by α5β1. The β3 cytoplasmic tail was also found to be necessary and sufficient for this regulation [122].

## 6. Is Integrin Crosstalk One of the Reasons for Integrin Targeting Therapy Failure?

Overexpressed integrins are potential drug and imaging targets. They are implicated in almost every step of cancer development and metastasis and, therefore, represent attractive targets for anticancer treatment. The literature on the cancer-promoting role of integrins is extensive and there are several cancer types in which integrin-targeting molecules, such as antibodies or integrin antagonists or inhibitors, have been tested in different clinical trial phases (for reviews, see [2,8,9,17,56,123]. Integrin signalling confers either primary or adaptive resistance of cancer cells to chemotherapy and radiotherapy [7,18,124] which are still the treatments of choice for many solid tumors. Therefore, different integrins remain interesting targets for the sensitization of tumor cells, or even cancer stem cells to chemotherapy and radiotherapy, but also inhibit metastasis, as it has been shown for breast carcinoma [64,125,126], head and neck squamous cell carcinoma [127], glioblastoma [128,129], melanoma [65,130], prostate cancer [131], ovarian cancer [132], lung cancer [133] and many others. Recent data have shown that integrin signalling confers resistance to targeted agents like vemurafenib [134] or lapatinib and trastuzumab [135]. Finally, not only integrins expressed on tumor cells, but also integrins expressed on cancer-associated fibroblasts (CAFs), namely α11β1, a receptor for fibrillar collagen during the differentiation of fibroblasts into CAFs, have an important role in the promotion of tumor growth and metastatic potential of non-small cell lung carcinoma cells. Specifically, the growth of A549 lung adenocarcinoma xenografts in integrin α11 knockout (−/−) mice was significantly impeded [136]. Therefore, integrins are potential targets for the enhancement of targeted therapy. Targeting integrins may be achieved with MoAbs, integrin antagonists and inhibitors, but also using RNA interference mechanism upon transfection with integrin specific small interfering RNA (siRNA). However, many factors prevent the development of integrin-based therapeutics for cancer. An important factor might be integrin crosstalk. Therefore, a deeper understanding of the mechanisms of integrin crosstalk may lead to the development of better integrin-based therapies or the development of integrin-related biomarkers that predict the success of therapy.

The clinical significance of integrin crosstalk is exemplified by the work from Dallari et al. [137] who showed that a changed number of available integrin subunits can be achieved by an integrin antagonist. They used natalizumab, a MoAb directed against integrin α4 in multiple sclerosis patients and reported upregulation of integrins α4 and β2 on monocyte subsets in the peripheral compartment. This is a clear example of how interfering with integrin expression/activation in patients can lead to integrin crosstalk. In one of the initial papers on integrin crosstalk, Blystone and colleagues [71] suggested caution in interpretation of the role of a particular integrin due to the fact that interfering with one subunit, or a heterodimer, could influence the expression or function of others. In accordance with this early notion, the relevance of integrin crosstalk in cancer therapy has become more recognized. The examples of integrin crosstalk found in the literature and summarised here, most of which were detected in tumor cells, either *in vitro* or *in vivo*, illustrates how targeting one integrin can have unwanted effects, like an increase in metastatic potential mediated by other integrin whose expression/activation is changed. A striking example of such an interplay has been demonstrated in an *in vivo* breast cancer mouse model in which β1 knockdown or exposure to β1-specific blocking antibody induces the expression of β3 leading to enhanced metastasis [88] or increased acinar cell growth and unchanged metastasts due to the β3 compensation [87].

## 7. Conclusions

Regarding the essential role of integrins in many cellular processes and their ability to direct the cell fate, it is of utmost importance that the mechanisms of their crosstalk are understood in detail. Since integrins are recognized targets in cancer treatment, integrin crosstalk needs to be taken into account and carefully analyzed in cancer cell models *in vitro* and *in vivo* to avoid unwanted effects. The question arises as to whether some of the integrin targeting agents in the clinics are failing, at least partly, because of the integrin crosstalk effect. This short review is our attempt to encourage researchers in the integrin field to pay attention to integrin crosstalk events with an ultimate aim of understanding its underlying mechanisms, cell biological significance, and potential implications for therapy and diagnosis.

## Figures and Tables

**Table 1:** Integrin crosstalk examples found in different research models. The table describes only integrin crosstalk events and corresponding outcome.

Cells	Intervention	Integrin Crosstalk	Outcome	Reference
MSC	lentivirus expressing α1 shRNA	↑ α2, α11 mRNA and protein expression	changes in cell adhesion and migration were not ascribed to changes in individual integrins α2 or α11 knockdown resulted in cell death	[72]
	lentivirus expressing α2 shRNA	↑ α1, α11 mRNA and protein expression		
	lentivirus expressing α11 shRNA	↑ α1 mRNA and protein expression		
MDA-MB-231	α3-blocking Ab	↑ α2β1 activation	↑ adhesion to COL	[93]
B10-renal cells from E18 α3-defficient mouse	*de novo* expression of human α3 subunit	↓ activation of αv	↓ cell adhesion to the α3 noncollagenous (NC1) domain of COL IV	[73]
A549, HT144, HUVEC	α3-blocking Ab	↑ αvβ3 activation	↑ adhesion to the α3 noncollagenous (NC1) domain of COL IV	
Wi26	microinjection of the α3 peptide representing the cytoplasmic domain of the α3 integrin	disengagement of the α6β1	reduced size of α6β1-focal adhesions	[94]
primary human skin fibroblasts	α3β1-function blocking Ab	↓ α6β1 integrin clustering	reduced size of α6β1-focal adhesions	[95]
CHO	stable transfection of murine α4 cDNA	↑ maturation of β1 precursor		[96]
Jurkat α4-defficient cells	*de novo* expression of α4	↑ αLβ2 activation	↑ migration that was αLβ2-dependent and VCAM-dependent	[75]
lymphocytes isolated from α4(S988A) bearing mice	↑ α4 activation (S988A) which precludes PKA-mediated α4 phosphorylation	↑ αLβ2 activation	↑ migration on ICAM-1 ↑ homing to B16 melanoma *in vivo*	[76]
Jurkat	crosslinking of α4β1	clustering αLβ2	↑ adhesion to ICAM-1	[74]
human T-cells	inhibition of α4β1 with blocking MoAb	α5β1 activation or expression was not analysed	↑ migration mediated by α5β1	[97]
	interaction of the αLβ2 with its ligand ICAM-1 or αLβ2-activation Ab	↓ binding of α4β1 and to a lesser extent α5β1	↓ adhesion mediated by α4β1 to FN and VCAM-1 and, to a lesser extent, α5β1 to FN	
HSC-3	α4-blocking Ab	↑ α5 and αv	↑ *in vitro* migration	[77]
	overexpression of α4 using transient transfection with plasmid containing α4 gene	↓ α5 and αv	↓ *in vitro* migration	
	α5-blocking Ab	↑ αv and α4	= *in vitro* migration	
	αv-blocking Ab	↑ α5 and α4	= *in vitro* migration	
CHO-B3 (negative for α5β1 and positive for αvβ3)	*de novo* expression of α5 using stable transfection with plasmid coding for α5 gene	↓ αvβ3 activation	↓ adhesion to FBG and αvβ3-mediated migration on FBG	[81]
U-251MG	ligation of α5β1 by plating cells on immobilised α5 MoAb	↑ αvβ3 activation	↑ αvβ3-mediated internalisation of VN	[80]
HUVEC	α5β1-blocking Ab and small molecule antagonists of α5β1	↓ αvβ3 activation	↓ formation of αvβ3 focal adhesions ↓ αvβ3-mediated *in vitro* migration on VN	[78]
chick CAM	α5β1-blocking Ab	↓ αvβ3 activation	↓ angiogenesis *in vivo*	
primary HUVEC	seeding to α5β1 selective substrate	↑ αvβ3 recruitment	↑ cell spreading	[79]
immortalized epidermal keratinocytes	lentivirus containing α6-specific shRNA	↓ α3 and α2 mRNA transcription and translation ↓ surface expression of α3β1 and α2β1	↓adhesion to LN332 and COL ↓migration	[98]
α9β1 *null* mice keratinocyte cell line	*de novo* expression of α9β1 using a retrovirus containing human α9 gene	↓ α3β1 activation	↓ α3β1-mediated migration *in vitro* on LN-332	[99]
CHO	αIIbβ3-specific inhibitor (ligand Ro43-5054)	↓ adhesive function of α5β1	↓ adhesion to FN	[29]
		↓ adhesive function of α2β1	↓ adhesion to COL	
HT29-D4	reduction of αv expression that is targeted to and degraded in lysosomes (reduces αvβ5/β6 expression)	↑ α2β1 activation	↑ cell migration	[100]
pKO, pan-ITG deficient murine fibroblasts reconstituted with αv and β1	binding to FN fragment FNIII7-10 (contains the RGD- and PHSRN-motifs)	αv integrins engagement activates α5β1 to establish additional adhesion sites to FN	↑ formation of α5β1 mediated adhesion clusters, adhesion strenghthening	[84]
HEK293 (β3wt) HUVEC MG-63	αvβ3-blocking Ab and αvβ3 ligand cyclic G-Pen-GRGDSPC-A (small peptide antagonist)	↓ α5β1 signallig	↓ α5β1-mediated migration toward FN, but not attachment to FN	[82]
K562	*de novo* expression of αvβ3 using stable transfection with plasmid coding for β3 gene	↓ α5β1 activation	↓ α5β1-mediated phagocytosis	[71]
TrHBMEC and HUVEC	αvβ3-blocking Ab	↓ α3β1 and α6β1 adhesion	↓ adhesion to LM5 and α4 LM G domain	[101]
	β1-blocking Ab	↓ αvβ3 adhesion	↓ adhesion to α4 LM G domain	
	α3 and α6-blocking Ab	↓ αvβ3 adhesion	↓ adhesion to α4 LM G domain	
MG-63	αvβ5-blocking Ab or cyclic peptide RGDfV	↑ α5β1 activation	unknown	[102]
CHO-B2 HeLa	deletion of α5β1 N-glycosylation site-11 that inhibits EGFR binding	↑ α6β4-EGFR complex	↑ cell proliferation	[103]
MG-63	knockdown of β1 using stable transfection with plasmid coding for shβ1 RNA	↓ protein maturation of α3 and α5 ↑ protein maturation of β1	=expression of surface β1	[104]
4T1	knockdown of β1 using stable transfection with plasmid coding for shβ1 RNA	↑ β3 mRNA	↓ *in vivo* tumor growth and ↑ *in vivo* metastasis which is not due to compensatory β3 expression	[88]
4T1 and MDA-MB-231	knockdown of β1 using stable transfection with plasmid coding for shβ1 RNA or exposure to β1-blocking Ab	↑ β3 mRNA and protein expression	↑ acinar cell growth, = growth of 3D organotypic culture and = *in vivo* metastasis due to compensatory β3 expression	[87]
HKC	knockdown of β1 using stable transfection with plasmid coding for shβ1 RNA	↑ αvβ3 activation	↑ αvβ3 localization to FA ↑ TGF-β1 induced COL mRNA expression	[85]
MMTV-NIC mice	mammary-specific deletion of β1 in the NIC model	↑ β3 mRNA and αvβ3 expression on the cell surface	modest delay of tumor onset and a significant inhibition of lung metastasis	[89]
MMTV-NIC mice	mammary-specific deletion of β1 in the NIC model	↓ β4; ↑ α5, αv, β3, β5 total protein levels measured by western blot	modest delay of tumor onset and a significant inhibition of lung metastasis	[86]
TrHBMEC	β1-blocking Ab	↓ αvβ3 activation	↓adhesion to a recombinant LM fragment and VN	[105]
GD25 (β1-*null*)	stable *de novo* expression of β1B isoform using stable transfection with plasmid coding for β1B gene	↓ αv adhesion	↓ αv containing focal adhesions and actin stress fibers ↓ spreading to FN (mediated by αvβ3)	[106]
GE11 β1-*null*	*de novo* expression of β1	↑ surface αvβ5	unknown	[107]
GD25 β1-*null*	*de novo* expression of the β1A or B	↓ β3 mRNA stability; ↓ surface αvβ3 ↑ surface αvβ5 (translational and post-translational level) = surface αv	unknown	[108]
PMN	β2-cross-linking Ab	↑ expression of β1 integrins on the cell surface	↑ adhesion to FN (through α5β1 and lesser extent α4β1) and COL (through α2β1)	[109]
	adhesion to COL gel (involvement of β2)	↑ expression of β1 integrins on the cell surface	↑ α2β1-dependent migration	
Jurkat	activation of β2 through the T-cell receptor or chemokines	↓ activation of α4β1	↑ *in vitro* migration ↓ binding to VCAM-1	[110]
MDA-MB-435S	transfection with β3 or β5-specific siRNA	↑ surface expression of αvβ5 or αvβ3, respectively, = surface expression of αv	= *in vitro* migration (unlike knockdown of αv which ↓ migration)	[64]
Cal27	*de novo* expression of β3 using stable transfection with plasmid coding for β3 gene	↑ β5 mRNA ↑ surface expression of αvβ5	unknown	[92]
HEp2	*de novo* expression of β3 using stable transfection with plasmid coding for β3 gene	↓ αvβ5 on the cell surface	↓ Adenovirus type 5 transduction efficacy	[90,91]
NIH3T3	deficient mutants β3^Y759A^ and β3^1-760^ that cannot bind protein kinase D1 (PKD1)	↑ α5β1 recycling and signalling	↓ peristent and directional migration ↑ random migration	[83]
immortalised fibroblasts from β3-null mice vs. WT fibroblasts	depletion of β3	↑ β1 activation	↓ peristent and directional migration ↑adhesion dynamics ↑migration speed	[111]
CHO	*de novo* expression of Tac-β3 constructs with impaired talin binding activity in αIIbβ3	↓ α5β1 activation	↓spreading on FBG	[112]
SW480	*de novo* expression of β6 using stable transfection with plasmid coding for β6 gene	↓ α2, α6, β1, β4 and β5 protein expression detected by mass spectrometry	↓ adhesion through β2, β3 and β4 ↓ adhesion to COL I and II, FN and VN ↑ *in vitro* invasion	[113]

↑, increase in the expression, or increase in the process stated; ↓, decrease in the expression, or the decrease in the process stated; =, no difference; FN, fibronectin; VN, vitronectin, COL, collagen; LM, laminin; FBG, fibrinogen; si, knockdown via small interefering RNA; sh, knockdown via shorth hairpin RNA; MMP, matrix metaloproteinase; KO, knockout; MoAb, monoclonal antibody; Ab, antibody. Cells: 4T1, mouse breast carcinoma; A549, lung carcinoma cells; Cal27, tongue squamous cell carcinoma; CAM, chorioallantoic membrane; CHO, chinese hamster ovary; GD25, mouse embryonic fibroblast cell line; GE11, β1-defficient epitheloid cells isolated from β1 chimeric embryos; HEK-293, embryonic kidney; HeLa, cervix adenocarcinoma; HEp2, laryngeal carcinoma; HKC, human renal tubular epithelial cell line; HSC-3, oral squamous cell carcinoma; HT-144, melanoma; HT29-D4, colon cancer; HUVEC, human umbilical vein endothelial cells; Jurkat, T cell lymphoma; K562, erythroleukemia; MDA-MB-231, triple negative breast carcinoma; MDA-MB-435S, melanoma; MG-63, osteosarcoma; MMTV-NIC mice, mouse mammary tumor virus activated erbB2 with an internal ribosome entry site driving expression of the Cre recombinase under the transcriptional control of the MMTV promoter; MSC, mesenchymal stem cells; NIH3T3, mouse fibroblasts; PMN, polymorphonuclear leukocytes; SW480, colorectal adenocarcinoma; TrHBMEC, immortalized human bone marrow endothelial cells; U-251MG, glioblastoma; Wi26, lung fibroblast line.

## Abbreviations

ECM	cell-extracellular matrix
IACs	integrin adhesion complexes
NAs	nascent adhesions
FAs	focal adhesions
FBs	fibrillar adhesions
RAs	reticular adhesions
HDs	hemidesmosomes
ER	endoplasmic reticulum
MoAbs	monoclonal antibodies
